# High Prevalence of Common Human Viruses in Thyroid Tissue

**DOI:** 10.3389/fendo.2022.938633

**Published:** 2022-07-14

**Authors:** Therese Weider, Angelo Genoni, Francesco Broccolo, Trond H. Paulsen, Knut Dahl-Jørgensen, Antonio Toniolo, Sara Salehi Hammerstad

**Affiliations:** ^1^ Department of Endocrinology, Morbid Obesity and Preventive Medicine, Oslo University Hospital, Oslo, Norway; ^2^ The University of Oslo, Faculty of Medicine, Oslo, Norway; ^3^ Department of Biotechnology, University of Insubria, Varese, Italy; ^4^ Department of Medicine and Surgery, University of Milano-Bicocca, Monza, Italy; ^5^ Department of Breast and Endocrine Surgery, Oslo University Hospital, Oslo, Norway; ^6^Department of Pediatric Medicine, Oslo University Hospital, Oslo, Norway; ^7^ Global Virus Network, University of Insubria, Varese, Italy; ^8^ The Specialist Center Pilestredet Park, Oslo, Norway

**Keywords:** autoimmune thyroid disease, Graves’ disease, Hashimoto’s thyroiditis, viral infection, enteroviruses, human herpesvirus 6, parvovirus B19

## Abstract

**Introduction:**

Evidence points to viral infections as possible triggers of autoimmune thyroid disease (AITD), but little is known about the prevalence of common viruses in the thyroid gland. Using a novel approach based on virus enrichment in multiple cell lines followed by detection of the viral genome and visualization of viral proteins, we investigated the presence of multiple human viruses in thyroid tissue from AITD patients and controls.

**Methods:**

Thyroid tissue was collected by core needle biopsy or during thyroid surgery from 35 patients with AITD (20 Graves’ disease and 15 Hashimoto’s thyroiditis). Eighteen thyroid tissue specimens from patients undergoing neck surgery for reasons other than thyroid autoimmunity served as controls. Specimens were tested for the presence of ten different viruses. Enteroviruses and human herpesvirus 6 were enriched in cell culture before detection by PCR and immunofluorescence, while the remaining viruses were detected by PCR of biopsied tissue.

**Results:**

Forty of 53 cases (75%) carried an infectious virus. Notably, 43% of all cases had a single virus, whereas 32% were coinfected by two or more virus types. An enterovirus was found in 27/53 cases (51%), human herpesvirus 6 in 16/53 cases (30%) and parvovirus B19 in 12/53 cases (22%). Epstein-Barr virus and cytomegalovirus were found in a few cases only. Of five gastroenteric virus groups examined, only one was detected in a single specimen. Virus distribution was not statistically different between AITD cases and controls.

**Conclusion:**

Common human viruses are highly prevalent in the thyroid gland. This is the first study in which multiple viral agents have been explored in thyroid. It remains to be established whether the detected viruses represent causal agents, possible cofactors or simple bystanders.

## Introduction

Autoimmune thyroid diseases (AITD), mainly comprising Hashimoto’s thyroiditis (HT) and Graves’ disease (GD), are the most common autoimmune endocrine disorders and affect 2-6% of the population (1). Predisposing genetic factors include HLA genes and other immunoregulatory genes (2–6). However, environmental factors are believed to play an important part as well. Viral infections may be one such environmental factor, and there is mounting evidence that viruses play a role in thyroid autoimmunity (7, 8).

Enteroviruses (9–11), parvovirus B19 (12–14) and hepatitis C virus (HCV) (15, 16) have all been associated with AITD. Among herpesviruses, Epstein-Barr virus (EBV) has been associated with GD (17–19), while human herpes virus 6 (HHV-6) and cytomegalovirus (CMV) may be associated with both HT and GD (20–24). The aforementioned viruses may produce subclinical disease and persistent infection at any age (25–29).

Case reports and autopsy studies show further associations of AITD with viral infections: rubella virus in congenital illness (30), human T cell leukemia virus (31), Hantaan virus (32), hepatitis E virus (33), HIV (34) and – more recently – the Severe Acute Respiratory Syndrome Coronavirus-2 (35–39). In addition, various gastroenteric viruses have been detected in children with other autoimmune disorders such as celiac disease and type 1 diabetes (40, 41).

We set out to investigate the presence of highly circulating viruses in a well-characterized and large collection of thyroid tissue samples. Following indications of literature, the presence of the following viruses were examined: enteroviruses, parvovirus B19, HHV-6, EBV, CMV, HCV and five gastroenteric viruses (adenovirus, astrovirus, norovirus, rotavirus and sapovirus). The small amount of biopsied thyroid tissue and the need for a wide spectrum of viral detection techniques capable of revealing a broad range of RNA and DNA agents were particularly challenging (42, 43). Results indicate that several common human viruses are frequently present in thyroid tissue from ATID patients and controls alike.

## Methods

### Study Participants, Collection of Thyroid Tissue and Blood Donors

We investigated a previously described collection of thyroid tissue samples stored at the Oslo University Hospital (11, 44). Fifteen HT samples were collected by core needle biopsy, while 20 GD samples were collected either by core needle biopsy or from surgical samples. Thyroid tissue samples from 18 patients undergoing neck surgery for reasons other than AITD (i.e. thyroid tumors or parathyroid adenomas) served as controls. Controls with positive anti-thyroid antibodies were excluded. Specimens were snap frozen in liquid nitrogen and stored at - 80°C. The Regional Ethics Committee approved the study (REK no. 1.2006.1950) and written informed consent was obtained from all participants. A group of blood donors (Varese, Italy) was introduced as a technical control for evaluating the prevalence of the investigated viruses in blood leukocytes of healthy subjects. The virology study was approved by the Ethics Committee of Ospedale di Circolo and Fondazione Macchi (Varese, Italy; 2018/02357094) and performed in accordance with the Declaration of Helsinki and local regulatory laws.

### Cultured Cell Lines Permissive for Enteroviruses and HHV-6

Five cell lines (AV3, RD, Ht-29, VC3 and HEK293) were obtained from The European Collection of Authenticated Cell Cultures and The American Type Culture Collection. These cell lines produce the main entry factors for enteroviruses (45, 46) and the two entry factors for HHV-6 (47, 48). Expression of virus receptors was evaluated by indirect immunofluorescence (IF) using the primary and secondary antibodies listed in **Table S1**.

Cell lines were cultured at 37°C in air with 5% CO2 using DMEM/F12 medium supplemented with L-glutamine, 7% heat-inactivated fetal bovine serum and penicillin/streptomycin. Cultures were checked monthly for mycoplasma contamination (MycoAlert ™PLUS Mycoplasma Detection Kit, Euroclone-Lonza, Pero, Italy).

### PCR Detection of Enterovirus and HHV-6 in Thyroid Tissue Following Enrichment in Cell Culture

**Figure 1:**
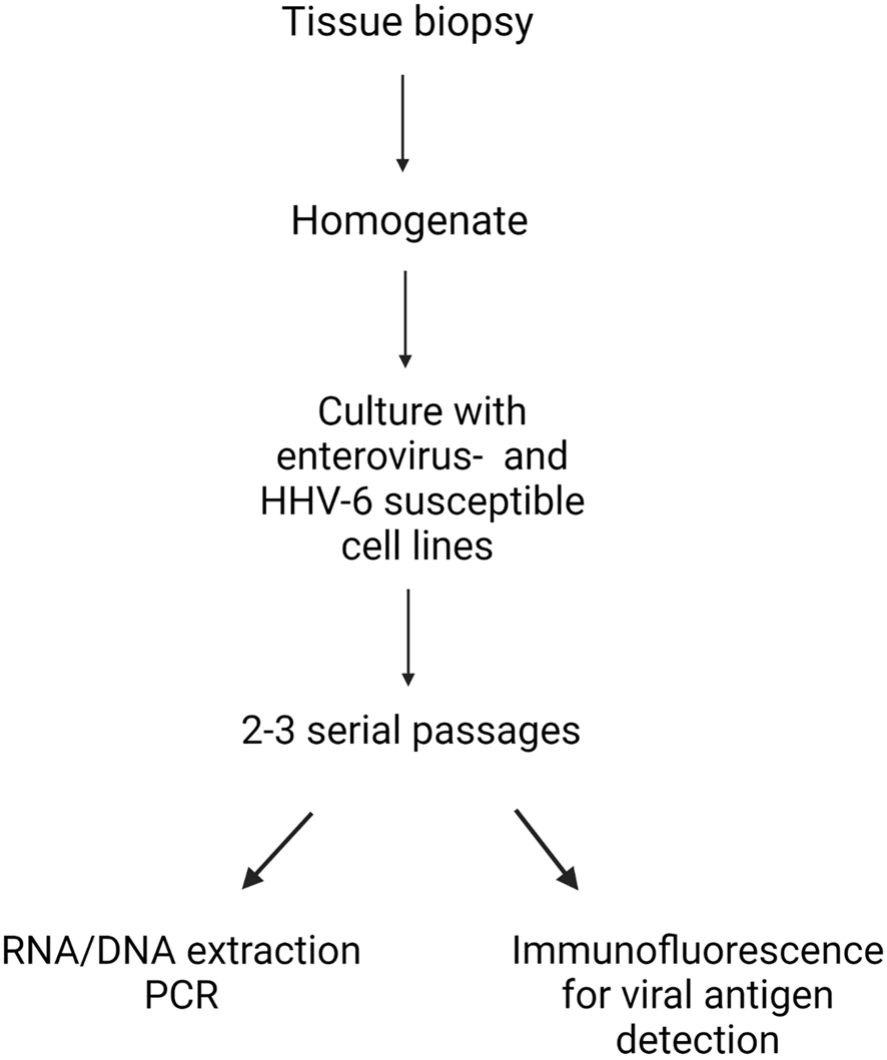
Model of the integrated procedure used for deriving virus strains from thyroid tissue.Thyroid biopsy tissues were made into homogenates, and then cultured with five cell lines with proven enterovirus- and human herpesvirus 6 (HHV-6) entry factors. After 2-3 serial passages, RNA (enterovirus) and DNA (HHV-6) were extracted, before viral genome detection by PCR. For each case, the expression of enterovirus and HHV-6 antigens in cultured cells were evaluated by immunofluorescence with a panel of enterovirus and HHV-6 antibodies. Created with BioRender.com.

Following published protocols (43, 49), thyroid homogenates were made in medium containing PANTA (a mixture of antibacterials and antifungals) using a FastPrep-24™ blender with glass beads (MP Biomedicals, Eschewege, Germany). Homogenates were cultured with the five cell lines described above using T25 flasks. After 2-3 serial passages, to detect viral genomes, DNA and RNA were extracted from each flask (supernatant plus cells) using an automated m2000sp instrument (Abbott Molecular, Rome, Italy). RNA was reverse transcribed to cDNA using SuperScript™ III Reverse Transcriptase and VILO™ Master Mix (Thermo Fisher Scientific-Invitrogen, Monza, Italy). End-point PCR assays for enteroviruses and HHV-6 were run in duplicates on Veriti™ Dx thermal cyclers (Applied Biosystems, Thermo Fischer, Monza, Italy) in a final volume of 50 μl using 15 μl of template. Five different sets of enterovirus-specific primer pairs targeting the 5’untranslated region of enterovirus genomes were used (**Table S2**). For HHV-6, we used pan-HHV-6 primers targeting a conserved sequence that comprise the cytoplasmic envelopment protein 2 - tripartite terminase subunit 3 (**Table S2**). PCR tests were deemed positive when an amplicon of the expected size was observed in the electropherogram. The viral nature of amplicons was verified by Sanger sequencing. Public databases were employed for attributing the sequences to the appropriate virus species. A schematic presentation of the method is provided (**Figure 1**).

### Antigen Detection of Enteroviruses and HHV-6 Antigens in Infected Cell Cultures

**Figure 2:**
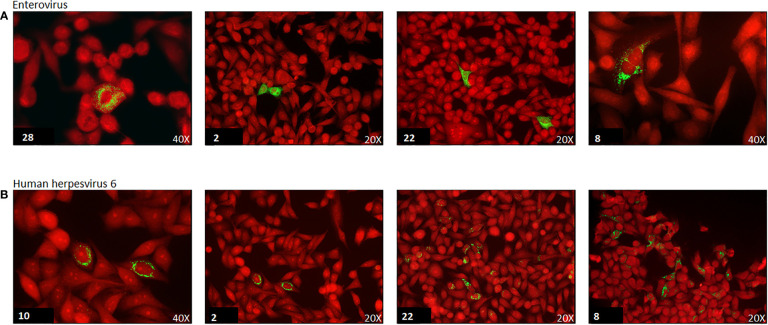
Immunofluorescence of enterovirus and human herpesvirus 6 (HHV-6) capsid protein in cell cultures of selected virus positive cases. Green immunofluorescence of cultured cell monolayers infected with enterovirus (panel **A**) or human herpesvirus-6 (panel **B**). Virus strains isolated from the thyroid of representative AITD cases. Case number is reported in the lower left corner. Red: Evans Blue counterstain. The original magnification is indicated (20x or 40x). Enterovirus staining of the VP1 capsid protein antigen by monoclonal antibody 6-E9/2. HHV-6 staining of the 140KD capsid polypeptide by monoclonal antibody F24H.

Cell cultures from the previously described five cell lines in Millicell^®^EZ slide 4-well glass (Merck-Millipore, Vimodrone, Italy) were incubated with supernatant from T25 flasks containing the five cell line mix exposed to thyroid tissue homogenates. After 3-4 days, cell monolayers were fixed in PBS containing 4% paraformaldehyde. Expression of enterovirus protein antigens was evaluated by IF with anti-EV monoclonal antibodies (mAbs) (**Table S1**) targeting either the VP1 capsid protein (mAbs 9D5, 6-E9/2, 5-D8/1) or the viral 3D RNA polymerase (mAbs 3D-02 and 3D-05; our own laboratory) (50). Enteroviral VP1 staining was deemed positive when granular cytoplasmic fluorescence was detected (**Figure 2**). HHV-6 antigens were detected with a mouse mAb directed at the 140 kDA capsid polypeptide.

### Whole Genome Amplification and qPCR for Viral Agents Other Than EVs and HHV-6

Since we searched for multiple viral agents, the small quantity of thyroid tissue posed a challenge. Thus, we resorted to whole genome amplification (WGA) technique for obtaining amounts of DNA templates sufficient for numerous PCR tests. The WGA2 method (Sigma-Aldrich, Milano Italy) was used according to the manufacturer’s protocol. Whereas DNA extracted from thyroid tissue was used directly in the WGA2 procedure, before WGA2 the RNA had to be converted into cDNA by reverse transcriptase (described above). qPCR assays were run in duplicate using 10 μl of WGA2-derived DNA template. A total volume of 25 μl was used for each PCR reaction run on ABI Prism 7500 thermal cyclers (Thermo Fischer-Applied Biosystems, Monza, Italy). **Table S3** shows the qPCR kits that have been used to detect CMV, EBV, parvovirus B19, HCV, adenoviruses F40-F41, astrovirus species 1-8, norovirus genogroups I and II, rotaviruses, sapoviruses. The analytical sensitivity of qPCR tests was 10-100 genome equivalents per reaction.

### Peripheral Blood Leukocytes From Blood Donors

Peripheral blood leukocytes from 19 healthy blood donors were used as technical controls. The investigated viruses were searched for in peripheral blood leukocytes of healthy persons since these cells represent a possible virus reservoir in persisting and latent infection. Leukocytes (including granulocytes) were obtained from a fresh aliquot of blood by centrifugation on discontinuous Ficoll-Hypaque gradients (density 1.077 and 1.119 g/ml). After washing 2x with medium, the leukocytes were cocultured with the aforementioned five cell line mix and processed as described above for detection of enterovirus and HHV-6. A leukocyte aliquot was preserved at -70°C for nucleic acids extraction, which was used for qPCR for viral agents other than enterovirus and HHV-6.

### Statistics

Demographic and clinical data of the investigated groups are presented as means with standard deviations or medians with interquartile ranges. The statistical significance of differences in mean was calculated using the T-test for normally distributed values in independent groups, or by the Mann-Whitney U test to compare non-normally distributed outcomes in independent groups. Results of virus tests are presented as numbers with percentages. The Pearson’s chi-square test was used to determine the statistical significance of differences in proportions. Analyses were performed using IBM SPSSS Statistics version 25. Statistical significance was set at p<0.05.

## Results

We investigated the presence of enteroviruses, parvovirus B19, HHV-6, EBV, CMV and HCV in addition to five gastroenteric viruses (adenovirus, astrovirus, norovirus, rotavirus and sapovirus) in thyroid tissue from AITD patients and controls. Patient characteristics are summarized in **Table 1**, and virus detection results are summarized in **Table 2**. Enteroviruses and HHV-6 were sought for using pre-enrichment of virus in cell culture before PCR and IF assays. As a technical control, the investigated viruses were searched for in peripheral blood leukocytes of 19 blood donors and were rarely detected: single, separated cases of EV, HHV-6 and EBV in three donors.

**Table 1:** Patient characteristics.

	Control group (n = 18)	Graves' disease (n = 20)	*P value*	Hashimoto's thyroiditis (n = 15)	*P value*
**Duration** *(months)*	–	12.0 (0.6-24.0)	*-*	0.0 (0.0-6.0)	*-*
**Female** *n(%)*	15 (83)	19 (95)	*0.247*	15 (100)	*0.102*
**Age** *(years)*	53.2±13.7	47.5±14.0	*0.209*	45.0±11.1	*0.072*
**TSH** *(mIU/L)*	1.6 (0.6-2.4)	<0.03 (<0.03-<0.03)	*<0.001*	3.3 (1.4-6.7)	*<0.01*
**FT4** *(pmol/L)*	13.3±2.5	27.6±12.8	*<0.001*	12.8±2.7	*0.589*
**TRAb** *(IU/l)*	<0.09	10.3 (3.6-18.8)	*<0.001*	<0.09	*-*
**TPO-Ab** *(kIU/L)*	<35	218.0 (61.0-408.0)	*<0.001*	927.0 (276.0-1500.0)	*<0.001*

FT4, free T4;TPO-Ab, thyroid peroxidase antibody; TRAb, thyrotropin receptor antibody. Reference ranges: TSH 0.5-3.6 mIU/l, FT4 8-20 pmol/l, TRAb <1,5 IU/l, TPO-Ab <35 IU/l.

P values compared to controls, and tested with Student's T-test, Mann-Whitney U test or Pearson's chi-square test.

Duration is the time from diagnosis to biopsy.

**Table 2:** Virus detection results for all 53 cases.

		Enteroviruses		Herpesviruses				PV-B19	HCV	Gastroenteric viruses				
Case no.	Group	PCR	IF	HHV-6 PCR	HHV6 - IF	EBV PCR	CMV PCR	PV-B19 PCR	HCV PCR	Adenovirus PCR	Astrovirus PCR	Norovirus PCR	Rotavirus PCR	Sapovirus PCR
1	GD	neg	neg	**POS**	**POS**	neg	neg	neg	neg	neg	neg	neg	neg	neg
2	GD	**POS**	**POS**	**POS**	**POS**	neg	neg	neg	neg	neg	neg	neg	neg	neg
3	GD	neg	neg	neg	neg	**POS**	neg	neg	neg	neg	neg	neg	neg	neg
4	GD	neg	neg	**POS**	**POS**	neg	**POS**	neg	neg	neg	neg	neg	neg	neg
5	GD	**POS**	**POS**	**POS**	**POS**	**POS**	neg	neg	neg	neg	neg	neg	neg	neg
6	GD	neg	**POS**	neg	neg	neg	neg	**POS**	neg	neg	neg	neg	neg	neg
7	GD	neg	**POS**	neg	neg	neg	neg	neg	neg	neg	neg	neg	neg	neg
8	GD	**POS**	**POS**	**POS**	**POS**	neg	neg	neg	neg	neg	neg	neg	**POS**	neg
9	GD	neg	neg	neg	neg	neg	neg	neg	neg	neg	neg	neg	neg	neg
10	GD	neg	neg	**POS**	**POS**	neg	neg	neg	neg	neg	neg	neg	neg	neg
11	GD	neg	neg	neg	neg	neg	neg	**POS**	neg	neg	neg	neg	neg	neg
12	GD	neg	**POS**	neg	neg	neg	neg	**POS**	neg	neg	neg	neg	neg	neg
13	GD	**POS**	**POS**	neg	neg	neg	neg	**POS**	neg	neg	neg	neg	neg	neg
14	GD	neg	neg	neg	neg	neg	neg	neg	neg	neg	neg	neg	neg	neg
15	GD	neg	neg	neg	neg	neg	neg	neg	neg	neg	neg	neg	neg	neg
16	GD	**POS**	**POS**	neg	neg	neg	neg	neg	neg	neg	neg	neg	neg	neg
17	GD	neg	neg	neg	neg	neg	neg	neg	neg	neg	neg	neg	neg	neg
18	GD	**POS**	**POS**	neg	neg	neg	neg	neg	neg	neg	neg	neg	neg	neg
19	GD	**POS**	neg	neg	neg	neg	neg	neg	neg	neg	neg	neg	neg	neg
20	GD	neg	neg	neg	neg	neg	neg	neg	neg	neg	neg	neg	neg	neg
21	HT	neg	**POS**	neg	neg	neg	neg	neg	neg	neg	neg	neg	neg	neg
22	HT	**POS**	**POS**	**POS**	**POS**	neg	neg	**POS**	neg	neg	neg	neg	neg	neg
23	HT	neg	**POS**	neg	neg	neg	neg	neg	neg	neg	neg	neg	neg	neg
24	HT	**POS**	**POS**	**POS**	**POS**	neg	neg	**POS**	neg	neg	neg	neg	neg	neg
25	HT	**POS**	**POS**	neg	neg	neg	neg	neg	neg	neg	neg	neg	neg	neg
26	HT	neg	neg	neg	neg	neg	neg	neg	neg	neg	neg	neg	neg	neg
27	HT	neg	neg	neg	neg	**POS**	neg	**POS**	neg	neg	neg	neg	neg	neg
28	HT	**POS**	**POS**	neg	neg	neg	neg	neg	neg	neg	neg	neg	neg	neg
29	HT	**POS**	**POS**	**POS**	**POS**	neg	neg	neg	neg	neg	neg	neg	neg	neg
30	HT	neg	**POS**	neg	neg	neg	neg	neg	neg	neg	neg	neg	neg	neg
31	HT	neg	neg	neg	neg	neg	neg	neg	neg	neg	neg	neg	neg	neg
32	HT	neg	neg	neg	neg	neg	neg	neg	neg	neg	neg	neg	neg	neg
33	HT	neg	neg	neg	neg	neg	neg	neg	neg	neg	neg	neg	neg	neg
34	HT	**POS**	**POS**	neg	neg	neg	neg	neg	neg	neg	neg	neg	neg	neg
35	HT	neg	**POS**	neg	neg	neg	neg	neg	neg	neg	neg	neg	neg	neg
36	Ctrl	**POS**	**POS**	**POS**	**POS**	neg	neg	neg	neg	neg	neg	neg	neg	neg
37	Ctrl	neg	neg	**POS**	**POS**	neg	neg	**POS**	neg	neg	neg	neg	neg	neg
38	Ctrl	neg	neg	neg	neg	neg	neg	neg	neg	neg	neg	neg	neg	neg
39	Ctrl	**POS**	**POS**	neg	neg	neg	neg	neg	neg	neg	neg	neg	neg	neg
40	Ctrl	neg	neg	**POS**	**POS**	neg	neg	neg	neg	neg	neg	neg	neg	neg
41	Ctrl	neg	neg	neg	neg	neg	neg	neg	neg	neg	neg	neg	neg	neg
42	Ctrl	neg	neg	neg	neg	neg	neg	**POS**	neg	neg	neg	neg	neg	neg
43	Ctrl	**POS**	**POS**	neg	neg	neg	neg	neg	neg	neg	neg	neg	neg	neg
44	Ctrl	neg	neg	**POS**	**POS**	neg	neg	neg	neg	neg	neg	neg	neg	neg
45	Ctrl	**POS**	neg	neg	neg	neg	neg	neg	neg	neg	neg	neg	neg	neg
46	Ctrl	neg	neg	**POS**	**POS**	neg	neg	**POS**	neg	neg	neg	neg	neg	neg
47	Ctrl	neg	**POS**	**POS**	**POS**	neg	neg	neg	neg	neg	neg	neg	neg	neg
48	Ctrl	neg	neg	**POS**	**POS**	neg	neg	neg	neg	neg	neg	neg	neg	neg
49	Ctrl	neg	neg	neg	neg	neg	neg	neg	neg	neg	neg	neg	neg	neg
50	Ctrl	neg	neg	neg	neg	neg	neg	neg	neg	neg	neg	neg	neg	neg
51	Ctrl	neg	neg	neg	neg	neg	neg	**POS**	neg	neg	neg	neg	neg	neg
52	Ctrl	**POS**	**POS**	neg	neg	neg	neg	**POS**	neg	neg	neg	neg	neg	neg
53	Ctrl	**POS**	**POS**	neg	neg	neg	neg	neg	neg	neg	neg	neg	neg	neg
Positive samples (n)		19	25	16	16	3	1	12	0	0	0	0	1	0
Positive samples (%)		**36%**	**47%**	**30%**	**30%**	**6%**	**2%**	**23%**	**0%**	**0%**	**0%**	**0%**	**2%**	**0%**

HHV-6 , human herpes virus 6; PV-B19 , parvovirus B19; EBV, Epstein-Barr virus; CMV, cytomegalovirus; HCV, hepatitis C virus.

Regarding enteroviruses, genomes were detected by PCR in 19/53 (36%) thyroid specimens. However, IF for the VP1 capsid protein proved more sensitive with 25/53 (47%) specimens being positive. In cells persistently infected by EVs, staining for the 3D RNA polymerase produced dotted fluorescence in the nuclear area (**Figure 2**). Twentyseven samples in total were enterovirus positive, with concordant PCR and IF results in 17 of the samples (63%). Enteroviruses were more frequent in AITD compared to controls, albeit not significantly: 67% in the HT group, 50% in the GD group and 39% in thyroid controls. Enterovirus sequences (relative to the partially conserved 5’UTR or to the VP4-VP2 genome regions) matched those of members of the A and B enteroviral species or - in a few cases – of the rhinovirus C species (**Table 3**). Regarding HHV-6, the PCR and IF methods were always concordant and positive in 16/53 cases (30%). The HHV-6 capsid protein antibody produced granular cytoplasmic fluorescence, often perinuclear (**Figure 2**). Regarding HHV-6, genome sequences refer to a strongly conserved tract (CEP2-TRM3). The sensitive PCR method and the antibodies used for HHV-6 detection did not allow discerning the A from the B species of the virus. The HHV-6 distribution did not differ significantly among the three investigated groups.

**Table 3:** RNA or DNA genome sequences representative of the enterovirus and HHV-6 strains obtained from the thyroid tissue of investigated cases.

Case no.	Virus genus	Partial sequence	Genome region	Best-matching virus sequence ^1^	Identities	Gaps
43 (CTRL)	Enterovirus	CTCAATCCAGGGGGTGGTGTGTCGTAATGGGCAACTCTGCAGCGGAACCGACTACTTTGGGTGTCCGTGTTTCCTTTTATTCTTATATTGGCTGCTTATGGTGAATTGCAATTACTGTTACCATATAGCTATTGGATTGGCCATCCAGTGACAAACAGAGCTTTGATATACTTGTTCGTGGGTTTCGTTCCACTCA	5'UTR	Coxsackievirus A6 (EV of the A species)	191/198 (96%)	3/198 (1%)
52 (CTRL)		TCCTAAGTTGCAAGCAGATGCCCTCAATCCAGGGGGTGGTGTGTCGTAACGGGCAACTCTGCAGCGGAACCGACTACTTTGGGTGTCCGTGTTTCCTTTTATTCTTACATTGGCTGATTATGGTGACAATTGAGAGATTGTTACCATATAGCTATTGGATTGGCCATCCAGTGATTCAACAGAGCATTGATATACCTGTTTGCGGGTTTTATTCCCACTCTCATCGTTCAGTCCATACTCTAAAGTACATTTTGATTCTGAACAATAGAAAATGGGCGCC	5'UTR	Coxsackievirus A6 (EV of the A species)	260/269 (97%)	2/269 (0%)
53 (CTRL)		CCACTTCAGGGGCCGGAAGAGTGACTAATCCGCATTCAGGGGGCCGGAGGGAATGATTTAAGCCGCATTTCAGAGGGCCGGAGAGAAGATTAGCCGCATTCAGGGGCCGGAGGAAG	5'UTR	Enterovirus A71 (EV of the A species)	45/53 (85%)	5/53 (9%)
34 (HT)		TGCGGAGCACAAGCCCTCAATCCAGGGGTTGGTGTGTCGTAATGGGCAACTCTGCAGCGGAACCGACTACTTTGGGTGTCCGTGTTTCCTTTTATTCTTATATTGGACTGCTTATGGTGACAATTGAGAGATTGTTACCATATAGCTATGGATTGGCCATCCAGTGACAAACAGAGCTTTGATATATTTGTTCGTGGGTTTCGTTCTACTCACCAGTCGTACAGTTCATACTTTAAAGTACATTC	5'UTR	Coxsackievirus A6 (EV of the A species )	239/246 (97%)	2/246 (0%)
36 (CTRL)		TAATTGGTAGTCCTCCGGCCCCTGAATGCGGCTAATCCTAACTGCGGAGCAGACACTCACAATCCAGTGAGCAGTCTGTCGTAATGGGCAACTCTGCAGCGGAACCGACTACTTTGGGTGTCCGTGTTTCCTTTTATTCTTATACTGGCTGCTTATGGTGACAATCA	5'UTR	Echovirus E19 (EV of the B species)	161/165 (98%)	0/165 (0%)
45 (CTRL)		TGCGGAGCAGACACCCACGAGCCAGTGGGCAGTCTGTCGTAATGGGTAACTCTGCAGCGGAACCGACTACTTTGGGTGT-CCGTGTTTCA	5'UTR	Echovirus E20 (EV of the B species)	83/89 (93%)	5/89 (5%)
18 (GD)		TCCTCCGGCCCCTGAATGCGCTAATCCTTACTGCGGAGCAGATACCCACAAACCAGTGGGCGGTCAGTCGTATCGGGCAACTCGGCGGCCGAACCGACTACTTTGGGTGTCCGTGTTTCCTTTTATTCCAAATATGGCTGCTTATGGTGACAATTGAAAGGTTGTTACCATATAGCTATTGGATTGGCCATCCGGTAACAAGCAGAGCGATTGTTTATCTGTTTGTTGGTTTCGTTCCATTAAATTATAAAGTT	5'UTR	Echovirus E6 (EV of the B species)	248/255 (97%)	1/255 (0%)
29 (HT)		CACAGATATTGCATGGAAGCCACCATGTGGATAGTCGTAACGGGCAACTGTGGGACGGAACCGACTACTTTGGGTGTCCGTGTTCCTTTTATTCCATTTATTATCTCATGGTGACAACGTATACAGATTATATGTTGTTCACCATGGGCGCTCAGGTATCAAAACAGAATACTGGCTCACATGAAAACGCAATCAATGCTAATAATGGAGGAGTGATCAAATATTTCAACATAAATTATTATAAGGACTCGG	5'UTR	Rhinovirus C (EV of the Rhinovirus C species)	249/253 (98%)	1/253 (0%)
16 (GD)		TCGTCCGTTCCCACAGTTGCCCGTTACGACTATTCCACATGGTGGCTTCCATGCAATTTTTCTGTGGGGTAGGATTATCCCGCATTCAGGGGCCGGAGGAAG	5'UTR	Rhinovirus C (EV of the Rhinovirus C species)	88/97 (91%)	5/97 (5%)
C19 (leukocytes of healthy blood donor)	HHV-6	GTAATTACAATCGACCATCAAAATATAAAGAGCACAGCACTTTTCGCCAGCTGCTACAATACACACGTAAGTACTATTAATTTAACTTTATTTTCAAAAATAAAAAATTAATACATAAAACAACACACAATTAGTCCATTACCACACGCTTACAA	CEP2-TRM3	HHV-6	154/154 (100%)	0/154 (0%)
CTRL cases: 44,36,46,47,37,40		GTAATTACAATCGACCATCAAAATATAAAGAGCACAGCACTTTTCGCCAGCTGCTACAATACACACGTAAGTACTATCAATTCAATTTTATTTTCAGAAACAAAAAATTAATACAGAAAATAACGCACACTTAGTCCATTACCACACGCTTACAA	CEP2-TRM3	HHV-6	154/154 (100%)	0/154 (0%)
GD cases: 1,12,2,4,5		CTAATTACAATCGACCATCAAAATATAAAGAGCACAGCACTTTTCGCCAGCTGCTACAATACACACGTAAGTACTATCAATTCAATTTTATTTTCAGAAACAAAAAATTAATACAGAAAATAACGCACACTTAGTCCATTACCACACGCTTACAA	CEP2-TRM3	HHV-6	153/153 (100%)	0/153 (0%)
HT cases: 28,22,29,24		CTAATTACAATCGACCATCAAAATATAAAGAGCACAGCACTTTTCGCCAGCTGCTACAATACACACGTAAGTACTATCAATTCAATTTTATTTTCAGAAACAAAAAATTAATACAGAAAATAACGCACACTTAGTCCATTACCACACGCTTACAA	CEP2-TRM3	HHV-6	153/153 (100%)	0/153 (0%)

¹Virus sequences attributed according to public databases (see **Table S1**).

5`UTR, 5`untranslated region; HHV-6, Human herpesvirus 6; CEP2, cytoplasmic envelopment protein 2; TRM3, tripartite terminase subunit 3; CTRL, controls; HT, Hashimoto's thyroiditis; GD, Graves' disease.

Parvovirus B19 – a virus producing a variety of clinical manifestations (51), including thyroid disorders (7, 12), was investigated using PCR amplification alone. Twelve out of 53 cases (23%) were positive. The detection rate between the three groups did not differ significantly.

Among the five taxonomic groups of gastroenteric viruses studied, a single GD case carried levels of a rotavirus strain close to the detection limit, indicating that gastroenteric viruses rarely infect the thyroid gland. EBV and CMV - two members of the herpesvirus group known to cause life-long latent infections in humans -were detected only in a few cases. Although the association of HCV with AITD has been demonstrated (15, 16), HCV was not detected in any of the investigated thyroid specimens.

The thyroid specimens were rarely devoid of viruses. Seventy-five percent of the investigated cases carried at least one virus in the gland, suggesting that unapparent viral infection of the thyroid is commonplace (**Table 4**). Concomitant infections with enteroviruses, HHV-6 and parvovirus B19, the viruses most frequently detected, were identified in several samples (**Table 5**). A single virus was found in 23/53 cases (43%), while 17/53 cases (32%) harbored two or three different agents. The viral load in thyroid tissue was consistently low, but some samples contained more virus that others. The inter-sample differences, however, were insignificant and could not be related to clinical status.

**Table 4:** Samples with single or multiple viruses detected.

	No virus	One virus	Two or more viruses	At least one virus

**All samples (n=53)**	13 (25%)	23 (43%)	17 (32%)	40 (76%)
**Controls (n=18)**	4 (22%)	8 (44%)	6 (33%)	14 (78%)
**Graves' disease (n=20)**	5 (25%)	8 (40%)	7 (35%)	15 (75%)
**Hashimoto's thyroiditis (n=15)**	4 (27%)	7 (47%)	4 (27%)	11 (73%)

**Table 5:** Concurrent virus detection.

	EV + HHV-6	EV+PV-B19	HHV-6+PV-B19

**All samples (n=53)**	8 (15%)	6 (11%)	4 (8%)
**Controls (n=18)**	2 (11%)	1 (6%)	2 (11%)
**Graves' disease (n=20)**	3 (15%)	2 (10%)	0 (0%)
**Hashimoto's thyroiditis (n=15)**	3 (20%)	2 (13%)	2 (13%)

EV, enterovirus; HHV-6, human herpes virus 6; PV-B19, parvovirus B19.

## Discussion

In this study, we investigated the presence of multiple viral agents in 53 thyroid tissue samples. To our knowledge, this is the first study to assess such a wide range of viruses in the thyroid. Results show that some human viruses are frequently present in the thyroid gland.

The *Enterovirus* genus contains more than 260 distinct virus types (52, 53). This makes the detection and distinction of the thyroid-infecting enterovirus types challenging. To increase the probability of virus detection, we increased the viral amount by enrichment in cell culture prior to searching for viral nucleic acids and antigens. Proof of live enteroviruses and HHV-6 in thyroid was obtained by: a) genome detection and sequencing as well as by b) visualization of viral proteins in cultured cells.

Enteroviruses were present in a high proportion of samples. The proportion of enterovirus-positive samples was slightly higher, albeit not significantly so, in the GD and HT group compared to controls. The majority of enterovirus-positive samples (63%) were positive by both PCR and IF assays. We previously published that the sensitivity of IF can be superior to PCR in the diagnosis of persistent infections (43), and analogous indications emerge from immunohistochemical studies (54). The huge variation of enterovirus genomes makes it impossible to match primer pairs with the enteroviral genomes of all genotypes. In contrast, the antigenic structure of the VP1 capsid protein, being more conserved, can be consistently detected by a small panel of pan-enterovirus antibodies.

By partial genome sequencing, the enterovirus strains found in thyroid appeared to belong to the A species (mainly Coxsackievirus A) or to the B species (likely echovirus types). However, the methods employed could not precisely identify the infecting virus genotype since this requires sequencing the VP1-VP2 enteroviral capsid region. This aim was not reached due to the minimal viral load present in the investigated thyroid samples (36, 43). Interestingly, surveillance and serology studies show that many different enterovirus types of the A and B species are circulating worldwide, often causing subclinical infection (55). More surprising is the finding of members of the rhinovirus C species within the thyroid. Notably, rhinoviruses are included in the *Enterovirus* genus (56, 57) and cause both the common cold and lower respiratory infections (58). A low-grade persistent enterovirus infection (not a latent one as in the case of herpesviruses) is linked to other autoimmune diseases, such as type 1 diabetes (40, 59–64). Experiments from our group showed that infection of cultured cells with enterovirus strains derived from the pancreas of type 1 diabetes cases enhance the expression of pro-inflammatory cytokines and chemokines associated with autoimmune disorders (65). In human thyroid, viral infection has also been shown to activate interferon signaling and the expression of interferon-stimulated genes (36, 49). Moreover, enhanced expression of HLA class I and of interferon-related STAT1 and PKR genes has been demonstrated in the thyroid tissue collection used in this study (9, 10).

HHV-6 comprises two species: HHV-6A which is more neurovirulent and associated with neuroinflammatory disorders (66) and HHV-6B that causes *exanthema subitum* (the sixth disease of infancy) (67). Unfortunately, the sensitive methods utilized in this study (PCR and IF) could not differentiate HHV-6A from HHV-6B. Thus, we may only conclude that an undetermined species of HHV-6 is frequently present in thyroid. Further research is needed in this regard since it has been suggested that HHV-6 of the A species is likely associated with HT and possibly with other autoimmune diseases (8, 21–23).

Among cases of coinfection, the association of enteroviruses with HHV-6 was the most frequent, followed by HHV-6 plus parvovirus B19, and enteroviruses plus parvovirus B19 (**Table 4**). It remains an open question whether carrying two or more viruses in the thyroid confers an increasing risk for AITD. It may be of interest to recall that HHV-6 transactivates EBV (68) and that the risk for multiple sclerosis increases when genetically predisposed persons become doubly infected with HHV-6A and EBV (69). Interestingly, a recent study shows that neonatal infection of mice with a roseolovirus related to HHV-6 may damage the thymus, thus disrupting central tolerance and causing autoimmunity later in life (70). Future studies may aim at evaluating whether similar dynamics may be operative in AITD.

Even though receptors for enterovirus, HCV and severe acute respiratory syndrome coronavirus 2, have been detected (9, 16, 71, 72), the entryway for viruses into the thyroid remains elusive. Since a wide range of viruses were detected, it seems reasonable to assume that there are multiple modes of entry to the thyroid. The majority of the investigated viruses (parvovirus B19, EBV, CMV, HHV-6 and enteroviruses) are transmitted *via* saliva and infect the mucosal epithelial cells of the oropharynx. The respiratory tract is in close proximity to the thyroid, thus one might speculate that respiratory viruses may enter the thyroid from the infected submucosal tissue. Moreover, the thyroid is highly vascularized, thus the blood stream could also be an entryway. Herpesviruses, like EBV, HHV-6 and CMV reside within host cells in a latent phase after primary infection (including granulocytes, lymphocytes and monocytes). EBV, HHV-6 and CMV are shed in the saliva, usually in the absence of symptoms, indicating that latency is a dynamic process and that reactivation may occur frequently, and usually in the absence of clinical disease or symptoms (73). Given the complexity of the herpesvirus life cycle, one explanation of the high prevalence of HHV-6 in thyroid tissue is fluctuating latent or low-grade infection in thyroid cells. The three viruses most frequently detected were enteroviruses of different species (51%) , HHV-6 (30%) and parvovirus B19 (22%). The seroprevalence of the two latter viruses in the adult population is high; approximately 70% for HHV-6 and 40-80% for parvovirus B19 (74, 75), thus, over their lifetime most people are being exposed to the above agents, in most cases without prolonged clinical signs. The high prevalence of infections by the latter agents may explain why the thyroid remains infected in some individuals.

Subacute thyroiditis (SAT) is a prevalent thyroid disease assumed to be of viral origin (76). Few authors have searched for virus within the thyroid gland in SAT, mainly due to the difficulty of justifying tissue biopsy or a needle aspirate in the absence of suspected neoplastic lesions. Evidence of virus from the few published studies is not conclusive, thus proving that viral etiology is hard to prove even though there is a strong clinical association of viral infection of the upper respiratory tract or influenza like malaise before the debut of SAT. Most people recover entirely from SAT, however, subsequent bouts of GD, persistent thyroid autoimmunity and a rise in thyroid autoantibodies occur (77–80).

Even though results show that the prevalence of the reported viral pathogens is not statistically different between AITD and thyroid controls, we do not believe that this upends the hypothesis that viruses may represent environmental triggers of thyroid autoimmunity. Actually, the findings may add evidence to this possibility proving that multiple viral agents are capable of producing unapparent infection of the gland. Moreover, genetic predisposition is a prerequisite for developing AITD. In genetically predisposed persons, susceptibility genes are expressed when the tissue undergoes microbial infection (81). The interplay of genetic predisposition and infectious insults could explain why common infections may give rise to autoimmunity in some but not in other individuals.

We recognize that criteria involving the detection of viral nucleic acids and proteins in diseased tissue cannot distinguish whether a virus is a causal agent, a causal cofactor or a simple bystander that homes to diseased tissue but does not contribute to pathology. However, the present study serves as a proof-of-concept and calls for further investigations of viral infections in the thyroid gland.

## Data Availability Statement

The original contributions presented in the study are included in the article/**Supplementary Material**. Further inquiries can be directed to the corresponding author.

## Ethics Statement

The studies involving human participants were reviewed and approved by The Regional Ethics Committee approved the study (REK no. 1.2006.1950) (Norway). The study was also approved by the Ethics Committee of Ospedale di Circolo and Fondazione Macchi (Varese, Italy; 2018/02357094). The patients/participants provided their written informed consent to participate in this study.

## Author Contributions

TW, AG, FB, and AT performed experiments, analyzed data, and drafted the manuscript. TP contributed to thyroid surgery and core needle thyroid sampling, and writing of the manuscript. SH contributed to all parts of the study, study design, clinical coordination and patient recruitment, data collection, analysis, and interpretation, and drafting of the manuscript. KD-J, as the principal investigator of the study, had the initial idea of the study and contributed to the study design, regulatory issues, international collaboration, data collection, analysis, and interpretation, and writing of the manuscript. KD-J and AT obtained funding.

TW, SH, and AT are the guarantors of this work and, as such, had full access to all the data in the study and take responsibility for the integrity of the data and the accuracy of the data analysis. Furthermore, this study has been performed in full adherence to ethical and legal requirements in Norway and Italy.

## Funding

South-Eastern Norway Regional Health Authority (HSØ grants to SH and KD-J) financed this work. For virus studies, funds have been obtained by the Juvenile Diabetes Research Foundation (JDRF grant 25-2012-770 to AT) and the Italian Ministry of Health (grant PE-2013-02357094 to AT). This research was performed with the support of the Network for Pancreatic Organ donors with Diabetes (nPOD; RRID: SCR_014641), a collaborative type 1 diabetes research project sponsored by JDRF (nPOD: 5-SRA-2018-557-Q-R). The content and views expressed are the responsibility of the authors and do not necessarily reflect the official view of nPOD. Organ Procurement Organizations partnering with nPOD to provide research resources are listed at http://www.jdrfnpod.org/for-partners/npod-partners.

## Conflict of Interest

The authors declare that the research was conducted in the absence of any commercial or financial relationships that could be construed as a potential conflict of interest.

## Publisher’s Note

All claims expressed in this article are solely those of the authors and do not necessarily represent those of their affiliated organizations, or those of the publisher, the editors and the reviewers. Any product that may be evaluated in this article, or claim that may be made by its manufacturer, is not guaranteed or endorsed by the publisher.
