# Supplementary material for: High Prevalence of Common Human Viruses in Thyroid Tissue
Source: Front Endocrinol (Lausanne). 2022 Jul 14;13:938633. doi: 10.3389/fendo.2022.938633 (PMC9333159; doi:10.3389/fendo.2022.938633)
Supplement: Supplementary file 1 [file DataSheet_1.docx]

*Cell lines, virus entry factor and reference virus strains.*

By indirect immunofluorescence, the AV3, RD, Ht-29, VC3 and HEK293 cell lines were evaluated for the expression of the major enterovirus cell entry factors: CAR; DAF; PVR; Integrin alpha-V/beta-3; Integrin alpha-V/beta-6 (KREMEN1); PSGL1; SCARB2; Heparan sulfate/Sialic acid; ICAM-1; ICAM-5/Telencephalin, as well as the two main HHV-6 receptors CD46 and CD134. The coxsackievirus B3 (CBV3, Nancy strain) and a blood isolate of HHV-6 (C19; Virology Laboratory, Varese, Italy) were used as reference for the detection of enterovirus and HHV-6 genomes, respectively.

*Detection of enterovirus and human herpesvirus 6 genomes by gene amplification*

Thermocycling parameters were as follows: a) enterovirus (touchdown PCR): (Stage 1 (x 1): 95°C, 5min; Stage 2 (x 10): 95°C 10sec, 64°C (then -1°C per cycle) 10 sec, 72°C 20 sec; Stage 3 (x30): 95°C 10sec, 55°C 10 sec, 72°C 20 sec; Stage 4 (x 1): 72°C, 5 min, 4°C); b) HHV-6 (Stage 1 (x 1): 95°C, 5min; Stage 2 (x 45): 95°C 10sec, 60°C 10 sec, 72°C 20 sec; Stage 3 (x 1): 72°C, 5 min, 4°C). PCR amplicons were identified by molecular size using a LabChip GX Touch 24 analyzer based on capillary electrophoresis (Perkin Elmer, Milano, Italy).

*Detection of enterovirus and human herpesvirus 6 antigens in infected cell cultures*

For additional virus typing, select monolayers were stained with monoclonal antibodies specific for coxsackieviruses group-B; echoviruses 4, 6, 9, 11, 30, 34; echoviruses 4, 6, 9, 11, 30; polioviruses 1-3 (Table S1). Alexa Fluor 488-goat anti-mouse IgG (Thermo Fisher Scientific, Monza, Italy) was used as secondary antibody. Slides were counterstained with Blue Evans. Images were taken with a Nikon E80i microscope and adjusted in brightness and contrast using Adobe Photoshop (Adobe, San Jose, CA, USA).

*Peripheral blood leukocytes of blood donors*

Leukocytes (including granulocytes) were obtained from a fresh aliquot of blood by centrifugation on discontinuous Ficoll-Hypaque gradients (density 1.077 and 1.119 g/ml). After washing 2x with medium, the leukocytes were cocultured with the human cell mix and processed as described above for virus detection. A leukocyte aliquot was preserved at -70°C for nucleic acids extraction.
